# SIRT1 inhibits rheumatoid arthritis fibroblast-like synoviocyte aggressiveness and inflammatory response via suppressing NF-κB pathway

**DOI:** 10.1042/BSR20180541

**Published:** 2018-06-21

**Authors:** Guoqing Li, Zhongbing Xia, Ying Liu, Fanru Meng, Xia Wu, Yuxuan Fang, Chunwang Zhang, Dan Liu

**Affiliations:** 1 Department of Rheumatology, Northern Jiangsu People’s Hospital affiliated to Yangzhou University, Yangzhou City, Jiangsu Province 225000, P.R. China; 2 Clinical Medical College, Dalian Medical University, Dalian City, Liaoning Province 116044, P.R. China; 3 Department of Pathology, Northern Jiangsu People’s Hospital affiliated to Yangzhou University, Yangzhou City, Jiangsu Province 225000, P.R. China

**Keywords:** fibroblast-like synoviocytes, inflammation, NF-κB, rheumatoid arthritis, sirtuin 1

## Abstract

Rheumatoid arthritis (RA) is an autoimmune disease of the joints characterized by synovial hyperplasia and chronic inflammation. Fibroblast-like synoviocytes (FLS) play a central role in RA initiation, progression, and perpetuation. Prior studies showed that sirtuin 1 (SIRT1), a deacetylase participating in a broad range of transcriptional and metabolic regulations, may impact cell proliferation and inflammatory responses. However, the role of SIRT1 in RA–FLS was unclear. Here, we explored the effects of SIRT1 on the aggressiveness and inflammatory responses of cultured RA-FLS. SIRT1 expression was significantly lower in synovial tissues and FLS from RA patients than from healthy controls. Overexpression of SIRT1 significantly inhibited RA-FLS proliferation, migration, and invasion. SIRT1 overexpression also significantly increased RA-FLS apoptosis and caspase-3 and -8 activity. Focusing on inflammatory phenotypes, we found SIRT1 significantly reduced RA-FLS secretion of TNF-α, IL-6, IL-8, and IL-1β. Mechanistic studies further revealed SIRT1 suppressed NF-κB pathway by reducing p65 protein expression, phosphorylation, and acetylation in RA-FLS. Our results suggest SIRT1 is a key regulator in RA pathogenesis by suppressing aggressive phenotypes and inflammatory response of FLS. Enhancing SIRT1 expression or function in FLS could be therapeutic beneficial for RA by inhibiting synovial hyperplasia and inflammation.

## Introduction

Rheumatoid arthritis (RA) is a chronic autoimmune disease characterized by synovial hyperplasia, inflammation and progressive destruction of the cartilage and bone, which ultimately lead to irreversible joint deformities and functional loss. The cause of RA is unclear, although many risk factors are recognized including genetics, environment, hormones, and lifestyle [1]. RA is a progressive disease that often develops systemic complications, including cardiovascular, pulmonary, skeletal, and psychological disorders. In general, RA patients are estimated to have a shorter life expectancy by as much as 10–15 years [2]. There is no cure for RA. Current treatment for RA relies on conventional and biologic disease-modifying antirheumatic drugs, which often lead to toxicity and only partial responses. Today, RA is one of the most common autoimmune disorders affecting 1% of the global population [3]. In China, nearly 5 million new RA cases are diagnosed each year [4], half of which progress into debilitating conditions within 10 years on onset, adding to significant socioeconomic costs. Therefore, the development of efficacious RA therapies remains an urgent public health need.

Fibroblast-like synoviocytes (FLS, also called type B synoviocytes) are a special type of mesenchymal-derived cells lining the internal synovium. FLS displays many markers of fibroblasts, including CD90, type IV and V collagens, and vimentin. FLS also shows characteristics distinct from other fibroblasts, including secretion of lubricin that lubricates the synovium, and expression of unique surface markers such as CD55, VCAM-1, cadherin-11, integrins, and their receptors. Studies from the past two decades established FLS as a prominent cellular participants in RA. RA–FLS directly participates in synovial hyperplasia and the production of cytokines that perpetuate local inflammation. RA–FLS also contributes to modulation of immune cells and proteolytic destruction of extracellular matrix, cartilage, and bone. Targeting RA-FLS has been recognized as a novel therapeutic approach with potentially improved clinical outcomes and less impact on systemic immunity [5].

Sirtuin 1 (SIRT1) is an NAD-dependent deacetylase engaging in a wide range of cellular functions such as transcription, cell cycle, DNA replication and repair, metabolism, apoptosis, and autophagy. SIRT1 is ubiquitously expressed and functions as a link between extracellular signals and transcriptional regulation of target genes. Several prior studies implicated a key role for SIRT1 in collagen-induced arthritis [6,7] and osteoclast differentiation [8–10]. However, the role of SIRT1 in RA-FLS has never been studied.

The objective of the present study is to systematically characterize the role of SIRT1 in RA-FLS, in order to provide insight for novel disease mechanisms and potential therapeutic targets for RA.

## Material and methods

### Preparation of human synovial tissues and FLS

Experiments involving human subjects were carried out in accordance with the declaration of Helsinki and were approved by the Medical Ethical Committee of Jiangsu University. Synovial tissues were obtained from 12 patients with RA (seven men and five women; 30–70 years old) during synovectomy or joint replacement surgery at Northern Jiangsu People’s Hospital affiliated to Yangzhou University. Healthy synovial tissues were collected from six knee joint trauma patients (three men and three women; 33–68 years old) during surgery and used as negative controls (NC). Signed informed consent was obtained from all participants. Synovial tissue samples were prepared as described previously [11]. FLS was obtained from synovial tissues by digesting with 2.5 g/l trypsin (Gibco, U.S.A.) with gentle agitation for 4  h at 37°C. FLS was cultured in Dulbecco’s Modified Eagle Medium (Gibco, U.S.A.) supplemented with 10% heat-inactivated fetal bovine serum (Gibco, U.S.A.), 100 U/ml penicillin, and 100 mg/ml streptomycin at 37°C and 5% CO_2_-enriched atmosphere. Cells between passages three to six were used for experiments. Visual examination of cell morphology under light microscopy and fluorescence activated cell sorting analysis of cells stained with anti-CD11b antibody (Santa Cruz Biotechnology, U.S.A.) confirmed that FLSs accounted for more than 95% of the cells (data not shown).

### Transfection

The SIRT1-overexpression vector (pCDNA3.1-SIRT1) and the blank vector pCDNA3.1 were transfected into RA-FLS cells using Lipofectamine 2000 (Invitrogen Life Technologies, U.S.A.) according to the manufacturer’s instructions. Cells were harvested at 48 h post-transfection for mRNA and protein expression measurements.

### Quantitative reverse transcription-PCR (RT-qPCR)

Total RNA was extracted using TRIzol reagent (Invitrogen Life Technologies, U.S.A.) according to the manufacturer’s instructions and reversely transcribed into cDNA using PrimeScript RT reagent kit (Takara Bio Inc., China). RT-qPCR was performed using the SYBR Premix Ex Taq kit (Takara Bio Inc.) on a 7500 fast real-time PCR System (Applied Biosystems, U.S.A.). The primers used for the RT-qPCR were as follows: SIRT1, forward 5′-TGG ACT CCA CGA CGT ACT-3′, reverse 5′-TCT CCT GGG AGG CAT AGA CC-3′; *GAPDH*, forward 5′–AGC CAC ATC GCT CAG ACA-3′, reverse 5′–TCT CCT GGG AGG CAT AGA CC-3′. For each sample, RT-qPCR were performed in triplicates, and the relative mRNA level was normalized to *GAPDH* using the 2^−ΔΔ*C*^_t_ method [12].

### Western blot

Total protein was extracted using RIPA lysis buffer (Sigma, U.S.A.) and concentration normalized using the BCA protein assay kit (Pierce, U.S.A). Equal amount of protein lysates (30 μg each lane) was resolved by 8–12% sodium dodecyl sulfate-polyacrylamide gel electrophoresis (SDS-PAGE) and transferred onto PVDF membranes (Bio-Rad, U.S.A.). The membranes were blocked in 5% nonfat milk in Tris-Buffered Saline with Tween-20 (TBST; Sigma, U.S.A.) and incubated with primary antibodies against NF-κB p65 (ab16502), phospho-NF-κB p65 (Ser536) (ab76302), acetyl-NF-κB p65 (Lys310) (ab19870), SIRT1 (ab110304), and *GAPDH* (ab8245) (all antibodies from Abcam, U.S.A.) overnight at 4°C, followed by incubation with peroxidase labeled anti-rabbit or anti-mouse secondary antibodies (Abcam, U.S.A.) at room temperature. The relative protein expression was detected using an enhanced chemiluminescent solution (Millipore, U.S.A.) and quantified by ImageJ using *GAPDH* as loading control.

### Proliferation assay

RA-FLS was seeded on 96-well plates at a density of 2 × 10^4^ cells/well and received pCDNA3.1-SIRT1 or pCDNA3.1 transfection at 100 ng/well. At 24, 48, and 72 h post-transfection, 10 μl/well Cell Counting Kit-8 (CCK-8; Sigma, U.S.A.) solution was added and incubated for 4 h at 37°C. The absorbance at 450 nm was measured on an ELx800 Absorbance Microplate Reader (BioTek, U.S.A.).

### Cell cycle and apoptosis assays

RA-FLS was detached as single-cell suspension 48 h post-transfection and fixed in 75% ethanol overnight at 4°C. Fixed cells were washed and stained with 25 mg/ml propidium iodide (PI; Sigma, U.S.A.) in PBS containing 0.1% Triton and 10 mg/ml RNase (Thermo Scientific, U.S.A.) on ice for 30 min in the dark. Cell cycle was analyzed by flow cytometry on a FACSCalibur system (BD Biosciences, U.S.A.).

For apoptosis assay, fixed and washed cells were detected using an annexin V-FITC apoptosis detection kit (BD Bioscience Pharmingen) and apoptosis rate was measured as the combined percentage of cells that underwent early apoptosis (FITC+/PI-) and advanced apoptosis and necrosis (FITC+/PI+). Activity of caspase-3 and caspase-8 was measured using colorimetric protease assay kits (Millipore, U.S.A.).

### Cell migration and invasion assays

Cell migration was determined by wound healing assay. Briefly, transfected cells were seeded on six-well plates and cultured to confluence. A wound was created by manually scraping the cell monolayer with a sterile 200 μl pipette tip. Cells were washed twice with PBS to remove floating cells and incubated in medium containing 1% FBS for 24 h. The wounds were imaged at two time points (0 and 24 h) with a phase-contrast microscope (Leitz, Germany) equipped with a Nikon DS-5M camera. Cell migration was calculated as percentage of healed distance relative to the initial wounds over 24 h.

Cell invasion was analyzed using Transwell invasion chambers (Corning, U.S.A.). Briefly, 3 × 10^4^ transfected cells in serum-free DMEM were added onto the upper chambers of 8-μm diameter Transwell inserts precoated with Matrigel (R&D Systems, U.S.A.), and 0.5 ml of DMEM with 10% FBS was added to the lower chamber. After 48 h incubation at 37°C, cells on the bottom chamber were fixed with 70% ethanol, stained with 0.1% Crystal Violet, and photographed under an inverted microscope. The numbers of cells in five random fields per well were counted and averaged as the invading cell number.

### Enzyme-linked immunosorbent assay (ELISA)

Cytokine levels in the supernatant of RA-FLS were measured 48 h post-transfection using ELISA kits for human TNF-α, IL-6, IL-8, and IL-1β (Genzyme Techne, U.S.A.) per manufacturer’s instructions.

### Statistical analysis

Results from at least three independent experiments were presented as mean ± standard deviation (SD). Statistical analysis was performed using the SPSS 19 software package (SPSS Inc., U.S.A.). Student’s *t*-test was used to evaluate the significance of difference between two groups. One-way analysis of variance followed by a post hoc Tukey’s test was used to test the significance of differences among three or more groups. *P*<0.05 was considered as statistically significant.

## Results

### SIRT1 was down-regulated in the synovial tissues and FLS of RA patients

**Figure 1:**
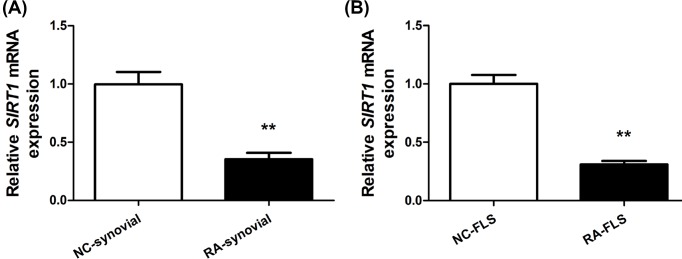
SIRT1 was down-regulated in the synovial tissues and FLS of RA patients *SIRT1* mRNA expression relative to *GAPDH* was measured by RT-qPCR in synovial tissues (**A**) and FLS (**B**) from healthy negative control subjects (NC, N = 6) and rheumatoid arthritis patients (RA, N = 12). Mean ± SD; **P < 0.01.

To test potential association between SIRT1 and RA, we first measured the expression level of SIRT1 mRNA in the synovial tissues and FLS obtained from 6 negative control individuals and 12 RA patients by RT-qPCR. Compared with NC, SIRT1 mRNA was significantly down-regulated in RA synovial tissues and in RA-FLS (Figure 1), implicating SIRT1 may potentially play a role in RA synovial tissues and FLS.

### SIRT1 inhibited RA-FLS proliferation

**Figure 2:**
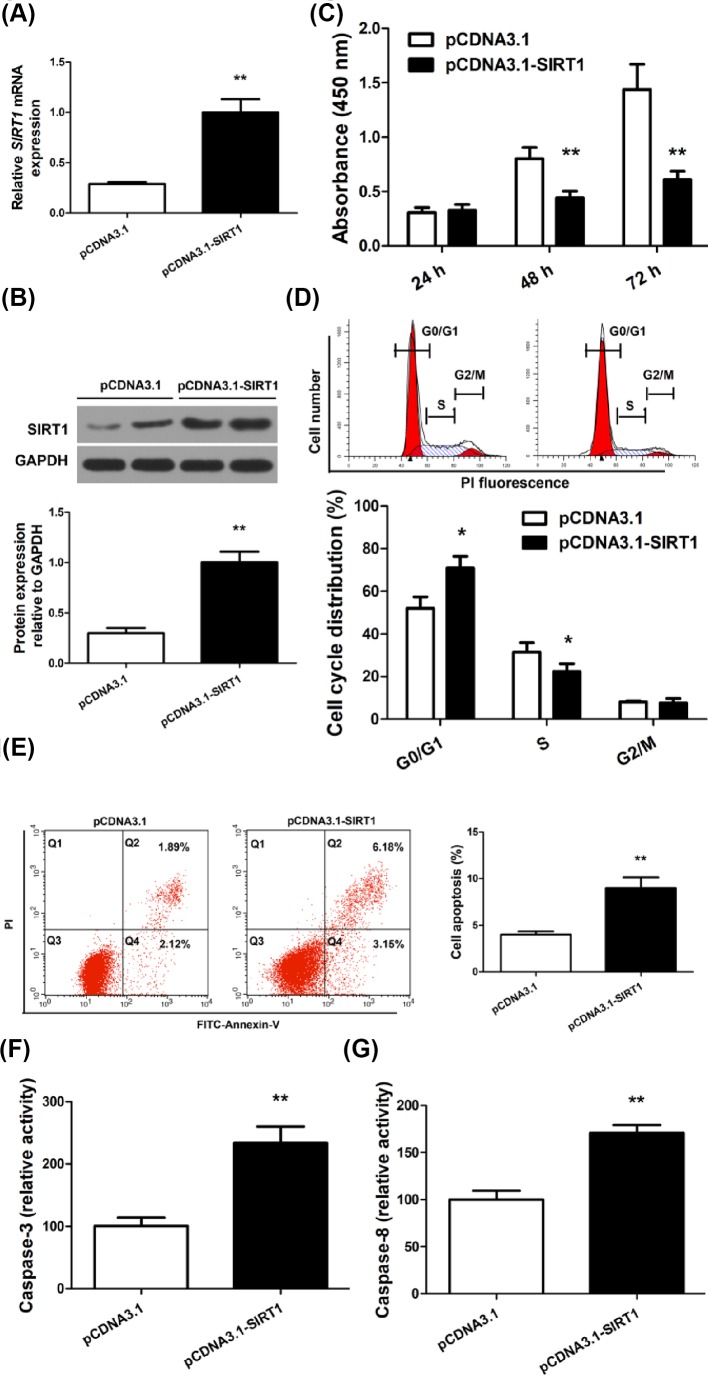
SIRT1 overexpression inhibited proliferation and induces apoptosis in RA-FLS RA-FLS was transfected with pCDNA3.1 or pCDNA3.1-SIRT1 and SIRT1 mRNA (**A**), and protein expression (**B**) relative to *GAPDH* was measured. (**C**) RA-FLS proliferation was measured by a CCK-8 assay at 24, 48, and 72 h post-transfection. (**D**) Cell cycle analysis of transfected RA-FLS was performed by flow cytometry after DNA staining with propidium iodide and categorized into G0/G1, S, and G2/M phases. (**E**) Apoptosis in transfected RA-FLS was measured by flow cytometry and quantified as combined percentage of FITC+/PI- (Q4) and FITC+/PI+ (Q2) cells. (**F** and **G**) Relative activity of caspase-3 and caspase-8 was determined by colorimetric protease assays in transfected RA-FLS; N = 6, mean ± SD, *P<0.05, **P<0.01.

In order to systematically study the role of SIRT1 in RA-FLS, primary RA-FLS culture was established using cells isolated from RA patients. Transfection of the SIRT1 overexpression vector significantly increased SIRT1 expression in RA-FLS at mRNA and protein level by more than 2-fold compared with the control vector (Figure 2A,B). To examine whether SIRT1 affects RA-FLS proliferation, we monitored proliferation of transfected RA-FLS for 72 h. Overexpression of SIRT1 markedly repressed RA-FLS proliferation at 48 and 72 h post-transfection, as evidenced by CCK-8 assay (Figure 2C) and by cell cycle arrest measured by flow cytometry (Figure 2D).

### SIRT1 induced RA-FLS apoptosis

Because proliferation assays only reflect viable cell numbers and do not distinguish the contribution from cell death, we then assessed the effects of SIRT1 on RA-FLS apoptosis. RA-FLS transfected with pCDNA3.1 or pCDNA3.1-SIRT1 was stained for apoptosis makers and analyzed by flow cytometry. SIRT1 overexpression increased the percentage of cells at both early apoptosis (Figure 2E, FITC+/PI-, Q4) and advanced apoptosis or necrosis (Figure 2E, FITC+/PI+, Q2). The overall apoptosis rate (the combined percentage of Q2 and Q4) showed a significant, nearly 2-fold increase in SIRT1-overexpressing RA-FLS (Figure 2E, right). Furthermore, to identify the pathways responsible for the amplified apoptosis by SIRT1, we measured the activity of caspases in transfected RA-FLS. SIRT1 overexpression led to more than 2-fold increase in caspase-3 activity (Figure 2F) and more than 1.5-fold increase in caspase-8 activity (Figure 2G). Together, these data suggested SIRT1 overexpression sensitized RA-FLS to apoptosis, partially by increasing intracellular caspase-3 and -8 activities.

### SIRT1 suppressed migration and invasion of RA-FLS

**Figure 3:**
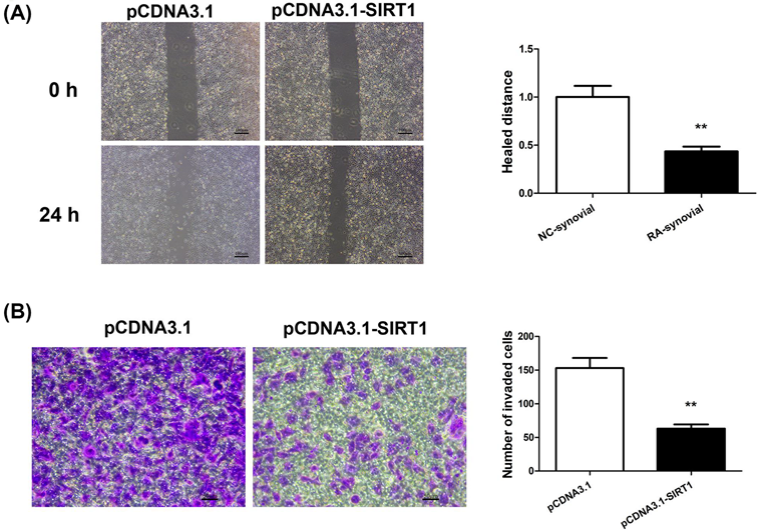
SIRT1 overexpression suppressed migration and invasion of RA-FLS RA-FLS was transfected with pCDNA3.1 or pCDNA3.1-SIRT1. The migration of transfected RA-FLS was measured by a wound healing assay (**A**), and the invasion was determined by a Transwell invasion assay (**B**). The representative micrographs of migrated cells over scratch wounds and invaded cells across Transwell inserts were shown in (A) and (B), and quantified respectively; N = 6, mean ± SD, **P<0.01.

SIRT1 has been shown to regulate migration and invasion of various cell types [13–16]. Since FLS migration and invasion are essential steps leading to joint destruction of RA, we assessed the effect of SIRT1 on these aggressive phenotypes of RA-FLS. An *in vitro* scratch wound healing assay revealed reduced migration in SIRT1-overexpressing RA-FLS as compared with empty-vector-transfected cells. The mean relative migration distance was decreased by nearly half in SIRT1-overexpressing RA-FLS compared with control cells (Figure 3A). In addition, cell invasion of pCDNA3.1- or pCDNA3.1-SIRT1-transfected RA-FLS was evaluated by a Transwell invasion assay. RA-FLS overexpressing SIRT1 displayed dramatic reduction in the number of invading cells across Transwell inserts (Figure 3B). Together, these data suggested SIRT1 overexpression impaired both the migratory and invasive ability of RA-FLS.

### SIRT1 reduced proinflammatory cytokine secretion by RA-FLS

**Figure 4:**
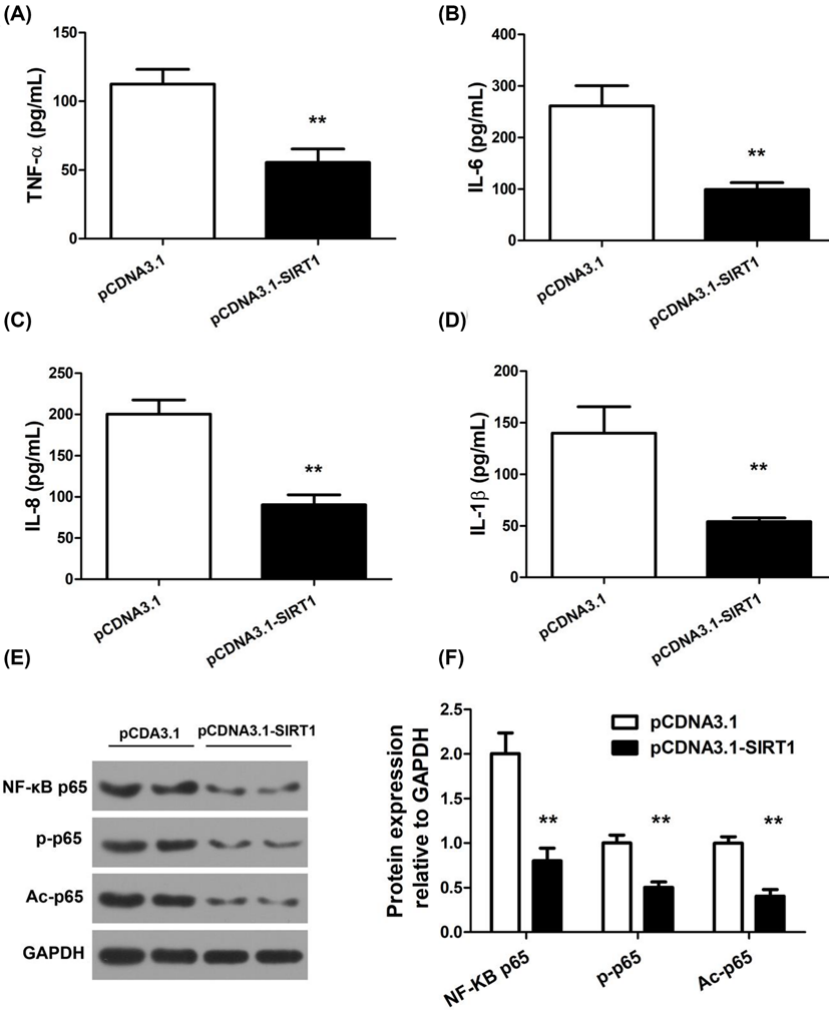
SIRT1 reduced proinflammatory cytokine production and NF-κB-related protein expression in RA-FLS RA-FLS was transfected with pCDNA3.1 or pCDNA3.1-SIRT1. TNF-α (**A**), IL-6 (**B**), IL-8 (**C**), and IL-1β (**D**) concentration in the supernatant of transfected cells were detected by ELISAs. Protein expression of NF-κB p65, p-p65 (Ser536), and Ac-p65 (Lys310) were detected by Western blot (**E**) and quantified using *GAPDH* as loading control (**F**); N = 6, mean ± SD, **P < 0.01*.*

In view of the fact that cytokines produced by RA-FLS play an important role in joint inflammation and damage [5], we hypothesized that SIRT1 may affect proinflammatory cytokine release by RA-FLS. We transfected RA-FLS with pCDNA3.1 or pCDNA3.1-SIRT1 and measured the levels of TNF-α, IL-6, IL-8, and IL-1β in the supernatant of cell culture medium by ELISAs. As shown in Figure 4A, substantial decrease in all tested proinflammatory cytokine levels was found in the supernatant of SIRT1-overexpressing cells.

### SIRT1 decreased expression and acetylation of NF-κB family proteins in RA-FLS

Based upon the inhibitory effects of SIRT1 on RA-FLS cytokine production, we further sought to investigate the molecular mechanism. To this end, we focused on the NF-κB pathway critical to synovial hyperplasia and inflammation. RA-FLS receiving pCDNA3.1 or pCDNA3.1-SIRT1 transfection was harvested for Western blot of NF-κB family proteins, including NF-κB p65, phospho-NF-κB p65 (p-p65, Ser536), and acetyl-NF-κB p65 (Ac-p65, Lys310). In RA-FLS, SIRT1 transfection significantly reduced the expression of NF-κB p65, p-p65, and Ac-p65 compared with the control transfection (Figure 4E,F).

## Discussion

RA is a chronic autoimmune disorder characterized by synovial hyperplasia, chronic inflammation, and progressive destruction of joints. FLS is a major cellular component that maintains the synovial homeostasis, and in the setting of RA, drives the synovial hyperplasia and inflammation progression. Emerging evidence indicate that inhibition of RA-FLS effector molecules might be beneficial for RA therapy [17]. The present study revealed SIRT1 as a potent inhibitor of RA-FLS function. Using FLS *in vitro* models derived from RA patients, we uncovered that, in addition to the suppression of RA-FLS proliferation, migration and invasion, SIRT1 also substantially reduced the proinflammatory cytokine production and proinflammatory NF-κB signaling in RA-FLS. Therefore, SIRT1 is a promising dual-effect target for both synovial hyperplasia and chronic inflammation in RA.

Synovial hyperplasia is characterized by increased proliferation and decreased apoptosis of RA-FLS. SIRT1 is NAD-dependent protein deacetylase that links transcriptional regulation to a variety of metabolic signals, such as nutrient deprivation, DNA damage, and oxidative stress [18]. One key downstream effect of SIRT1 is the regulation of cell proliferation. However, the exact effect of SIRT1 on cell proliferation has been inconsistent and appears to be cell type specific. For example, SIRT1 increased skeletal muscle proliferation [19], but inhibited proliferation of endothelial cells and certain cancer cell types [20–22]. We found in RA-FLS, SIRT1 overexpression significantly inhibited cell proliferation and arrested cell cycle, consistent with previous reports showing SIRT1 as an inhibitor against cell proliferation in certain proliferative diseases such as cancer [20,23–26]. One way to reconcile the opposing effects of SIRT1 on the proliferation of different cells is that certain proliferative tissues including RA-FLS and a subset of cancers may share common SIRT1-dependent signaling pathways distinct from those in other cell types. This notion is supported by several lines of evidence. First, in RA synovium, FLS is exposed to metabolic stress signals commonly shared by cancer cells, such as hypoxia and elevated local inflammatory cytokines. Second, compared with FLS in normal synovium, RA-FLS displays altered phenotypes that are akin to cancerous cells [5]. Third, SIRT1 is down-regulated in both RA synovial tissues and RA-FLS as shown by our data, similar to its down-regulation in certain cancers as shown by others [27], an effect that could be explained by common SIRT1-dependent signaling pathways in these cell types. Fourth, in cancer cells, SIRT1 overexpression induced a G1-phase cell cycle arrest via cyclin D1 signaling [27], an effect that is in line with our finding on RA-FLS cell cycle arrest.

The effect of SIRT1 on RA-FLS apoptosis has been elusive. Several prior studies showed that SIRT1 down-regulated proapoptosis protein CYR61 [28] and protected against apoptosis in RA-FLS [29,30], whereas others demonstrated SIRT1 activation by resveratrol increased RA-FLS apoptosis [31]. The disparity of effect on apoptosis of the same cell type appeared to be associated with SIRT1 subcellular localization. Recent studies showed the antiapoptotic effect of SIRT1 was associated with its nuclear localization, and the proapoptotic effect was associated with its cytoplasmic localization [32,33], and the shuttling between the nucleus and cytoplasm was a regulatory mechanism of SIRT1 that was tightly controlled and highly responsive to developmental stages and environmental signals. Our results demonstrated SIRT1 transient overexpression significantly increased RA-FLS apoptosis, and therefore, may offer novel approaches against synovial hyperplasia. Further investigation is required to determine if SIRT1 cytoplasmic localization is necessarily associated with the proapoptotic effect on RA-FLSs, what extracellular signals are required, or whether additional unknown mechanism is instead responsible for the proapoptotic effect of SIRT1.

Chronic synovial inflammation is a hallmark of RA. Activated RA-FLS contributes to synovial inflammation by secreting proinflammatory cytokines that serve as kindling for an immune response. These cytokines in turn recruit and activate leukocytes to the RA synovium, amplifying local inflammation and tissue damage. Cytokines not only are responsible for the inflamed joints but also have profound systemic effects. Therefore, cytokines represent novel therapeutic opportunities at both local and systemic levels. Several biologic agents targeting TNF-α and IL-1 are already licensed for RA treatment, and others showed promise in clinical trials [17]. Unfortunately, to date, no single biological agents or inhibitor therapy specifically targeting one cytokine can cure disease or achieve complete remission in RA patients. Considering the high redundancy in the production and action of cytokines, one alternative inhibitory strategy is to target their common intracellular signaling mechanisms. Our results showed SIRT1 potently suppressed several known proinflammatory cytokines secreted from RA-FLS, supporting the view that SIRT1 could act upon the common regulatory machinery of a broad array of inflammatory mediators in RA. Therefore, SIRT1 may represent an alternative therapeutic target that could potentially offer better efficacy in inhibiting RA-FLS-derived cytokines.

NF-κB is constitutively activated in RA and maintains a proinflammatory, proliferative, and damaging phenotype of RA-FLS [5,34,35]. NF-κB family contains five proteins: RelA (p65), RelB, c-Rel, NF-κB1 (p105/p50), and NF-κB2 (p100/p52). Among them, p50 and p65 are the most relevant subunits to RA synoviocytes by immunohistochemistry [5]. Prior studies showed that suppression of NF-κB p65, the main effector subunit of the classical NF-κB pathway, protects against RA [36]. The transcriptional activity of p65 could be further regulated by phosphorylation and acetylation. For instance, p65 Ser536 phosphorylation contributes to the inhibition of p53 transcriptional activity and antiapoptotic effects [37,38], and p65 acetylation at Lys310 is required for its full transcriptional activity [39]. Our results showed that SIRT1 overexpression significantly decreased NF-κB-p65 expression, p65 phosphorylation, and acetylation in RA-FLSs, supporting the view that SIRT1 serves as a master regulator of the NF-κB pathway, coordinating multiple downstream signals that collectively reduce synovial inflammation.

To summarize, SIRT1 suppressed RA-FLS proliferation, migration and invasion, induced apoptosis and reduced proinflammatory cytokine secretion from RA-FLS. We found these protective effects were partially due to SIRT1 inactivation of the NF-κB pathway. Our findings have therapeutic significance, because the SIRT1-mediated effects on RA-FLS involve a dual mechanism targeting both synovial hyperplasia and inflammation. Novel approaches aiming at augmenting the protective effect of SIRT1 may therefore be a promising option for FLS-targeted therapy in RA.

## Abbreviations

CCK-8: Cell Counting Kit-8
ELISA: enzyme-linked immunosorbent assay
FBS: fetal bovine serum
FLS: fibroblast-like synoviocytes
NC: negative controls
PI: propidium iodide
RA: rheumatoid arthritis
RT-qPCR: quantitative reverse transcription–PCR
SD: standard deviation
SDS-PAGE: sodium dodecyl sulfate–polyacrylamide gel electrophoresis
SIRT1: sirtuin 1
TBST: Tris-buffered saline with Tween-20
